# Sudden death due to gas gangrene caused by *Clostridium septicum* in goats

**DOI:** 10.1186/s12917-018-1747-y

**Published:** 2018-12-18

**Authors:** Abdullah Gazioglu, Burcu Karagülle, Hayati Yüksel, M. Nuri Açık, Hakan Keçeci, Muhammet Bahaeddin Dörtbudak, Burhan Çetinkaya

**Affiliations:** 1 Department of Veterinary Science, Vocational School of Technical Sciences, University of Bingol, 12000 Bingol, Turkey; 2 Department of Microbiology, Faculty of Veterinary Medicine, University of Firat, 23119 Elazig, Turkey; 3 Department of Pathology, Faculty of Veterinary Medicine, University of Bingol, 12000 Bingol, Turkey; 4 Department of Microbiology, Faculty of Veterinary Medicine, University of Bingol, 12000 Bingol, Turkey; 5 Department of Internal Medicine, Faculty of Veterinary Medicine, University of Bingol, 12000 Bingol, Turkey

**Keywords:** *Clostridium septicum*, Goat, Gas gangrene

## Abstract

**Background:**

Even though gas gangrene caused by *Clostridium septicum* in goats is mentioned in the classical textbooks, we have not managed to find any case description in the literature.

**Case presentation:**

Clinical signs resembling gas gangrene such as subcutaneous bloating, edema and crepitation were detected at various body parts of nine pregnant animals at the ages of 2–3 years on a hair goat farm (*n* = 170) located in Bingol province, Eastern Turkey. Five of these suspected animals with severe clinical symptoms died within 2 days. Various samples such as internal organs, edematous skin and edema fluid collected from dead and live animals were analyzed for the presence of clostridial agents by histopathological and microbiological methods. As a result of macroscopic and microscopic examination, lesions of gas gangrene were detected. The suspected isolates were identified and confirmed as *C. septicum* by bacteriological and molecular methods.

**Conclusion:**

The present study was the first to report identification of *C. septicum* as primary agent in the gas gangrene of goats.

## Background

Gas gangrene, previously defined as malignant edema, is an important clostridial infection worldwide. It is characterized with necrosis in soft tissues, especially in muscle, toxemia, and sudden deaths in animal species, particularly ruminants and humans [1, 2]. Several clostridial agents including *Clostridium septicum*, *C. sordellii*, *C. chauvoei*, *C. perfringens* type A and *C. novyi* type A have been linked with the etiology of gas gangrene [3, 4]. Although information on the pathogenesis of the disease is limited, it is considered that vegetative or spore forms of at least one *Clostridium* species cause exogenous disease in animals by entering the body through abraded skin such as scratches, cuts and castration wounds [5]. Despite the fact that tissue traumas play an important role in the growth of *Clostridium* agents in soft tissues, especially in small ruminants, *C. septicum* can easily grow in these regions without tissue trauma as it is more aero-tolerant than other species [6].

*C. septicum* is the etiological agent of suppurative and necrotizing abomasitis (known as braxy) in ruminants including sheep and calves. This agent is also responsible for gas gangrene in cattle*,* sheep, horses and other species, and gangrenous dermatitis and cellulitis in poultry [4, 7, 8]. Extracellular toxins produced by *C. septicum* such as deoxyribonuclease, hyaluronidase, neuraminidase and alpha toxin play significant roles in the occurrence of the disease. While some of these toxins are involved in the spread of bacterium within the body, alpha toxin plays a major role in the formation of tissue necrosis [9, 10].

Even though gas gangrene caused by *C. septicum* in goats is mentioned in the classical textbooks [11], we have not managed to find any case description in the literature. In the present study, tissue and organ samples collected from a goat herd suspected of gas gangrene were examined for the presence of *C. septicum* by conventional and molecular methods.

## Case presentation

Clinical signs resembling gas gangrene such as subcutaneous edema and crepitation were detected at various body parts of nine pregnant animals (last month of pregnancy) at the ages of 2–3 years on a hair goat farm (*n* = 170) located in Bingol province, Eastern Turkey. The degree of clinical signs was observed to be more severe (impaired general condition, skin lesions on all legs) in five animals which died one after another within 30 h. No attempt could be made for the treatment of these animals because they were at the terminal stage of the disease when we were alert to the problem. On the other hand, the remaining four animals had a milder course of disease which suggested that they were at the onset of the disease. These animals were treated with antibiotics (Penicillin G 8.000 IU/kg + Streptomycin 10 mg/kg, s.i.d. IM, for 3 days) after collecting the required samples and the signs of gas gangrene disappeared within 3 weeks following antibiotic therapy.

Systemic necropsies were performed on dead animals within 2 h at the latest. Following necropsy, samples taken from subcutaneous fascia, lungs, mesenteric lymph nodes, cardiac muscle and kidney tissue were fixed in 10% buffered formaldehyde solution. Paraffin blocks were prepared and cut to a thickness of 5 μm from tissues taken for routine histological follow-up. The prepared paraffin sections were stained with hematoxylin-eosin and evaluated histopathologically on a light microscope (Leica, Germany).

Samples were collected in sterile conditions from edematous skin lesions and internal organs of dead animals for microbiological analyzes. In addition, edema fluid was obtained with sterile syringes from the edematous skin area while the animals were alive. The samples were transported within 2 h under cold chain conditions (+ 2/+ 8 °C in icebox) to the laboratories where culture procedures were carried out. Homogenized internal organs and edema fluid were first subjected to enrichment in Cooked Meat Medium (Oxoid) for anaerobic isolation. One ml of edema fluid was added to the bottom of a 10 ml Cooked Meat Medium with a sterile Pasteur pipette and incubated in an anaerobic jar for 24 h at 37 °C. Anaerobic media were supplied with anaerobic gas kits (Anaerocult A, Merck). In addition, samples were incubated in 5% blood agar for 24 h at 37 °C for aerobic microorganism isolation. In the anaerobic conditions, turbidity was seen at the bottom of the Cooked Meat Medium tube. For this reason, 100 μl of Cooked Meat Medium was inoculated in 5% blood agar and incubated for 24 h at 37 °C in anaerobic conditions.

DNA samples extracted from the suspected colonies by conventional Phenol extraction method were subjected to Polymerase Chain Reaction (PCR) combined with a pair of species-specific primers derived from flagellin gene (*fliC*) region of *C. septicum* [12]. Due to the fact that *C. chauvoei* has similar biochemical characteristics and it has been isolated together with *C. septicum* in many cases, the growth colonies were also examined for the presence of *C. chauvoei* by a species specific PCR [12]. The presence of alpha toxin, a lethal virulence factor which plays a significant role in the pathogenesis of gas gangrene caused by *C. septicum*, was also investigated in the current study by employing a PCR combined with alpha toxin (Hemolysin) gene [13]. The primers and PCR conditions used in this study were presented in Table 1. In order to confirm PCR findings, one-way DNA sequence analysis was performed on two randomly selected samples from PCR products of the flagellin gene region (Sentegen Biotech Laboratory, Ankara, Turkey).

**Table 1:** Primers and PCR conditions used in this study

Target gene	Sequences (5′-3′) (amplicon sizes)	PCR conditions	References
*C. septicum* flagellin gen (*fli*C)	FlaF- AGAATAAACAGAAGCTGGAGATG FlaseR-TTTATTGAATTGTGTTTGTGAAG (Amplicon:294 bp)	94 °C 1 min 55 °C 1 min 72 °C 90 s (30 cycles)	[12]
*C. chauvoei* flagellin gen (*fli*C)	FlaF- AGAATAAACAGAAGCTGGAGATG FlachR-TACTAGCAGCATCAAATGTACC (Amplicon:535 bp)		
Alpha toxin Gen	F-AATTCAGTGTGCGGCAGTAG R-CCTGCCCCAACTTCTCTTTT (Amplicon:270 bp)	94 °C 1 min 55 °C 1 min 72 °C 1 min (35 cycles)	[13]

In the detailed clinical examination, poor body condition, depression, cyanosis in the eye conjunctiva, tachypnea, tachycardia and hard vesicular sounds in lung auscultation were observed in all animals with severe disease. The inner side of the affected leg was gangrenous which resulted in lameness. In addition, crepitated gas formation was observed in the palpation of the affected skin area and abundant yellow-red liquid was seen at subcutaneous tissue following puncturing.

**Fig. 1:**
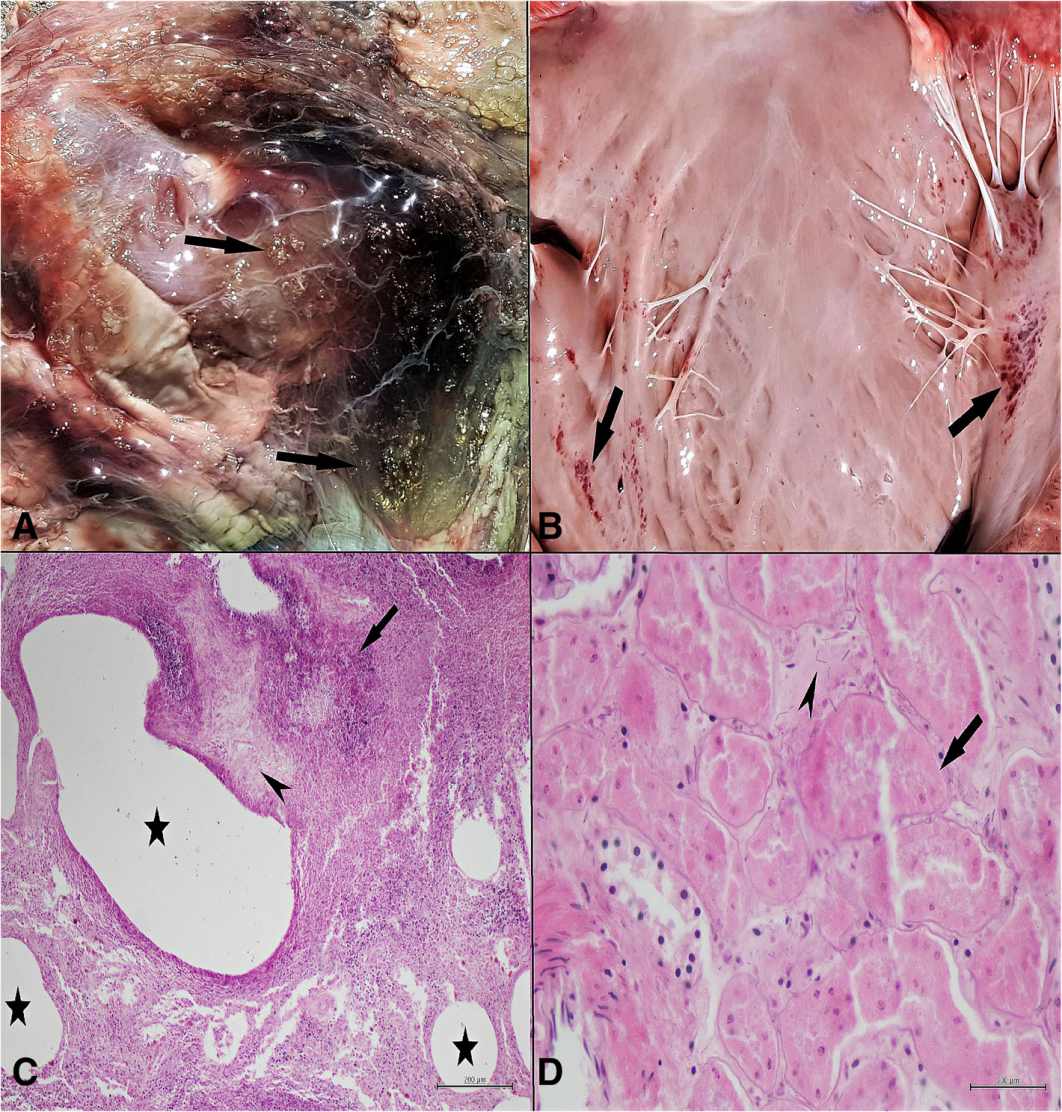
Macroscopic and microscopic results obtained from various tissue and organ samples. **a** Macroscopically, greenish appearance containing bubbles in the subcutaneous fascia (arrows). **b** Petechial hemorrhages in the endocardium (arrows). **c** Microscopically, focal necrosis (arrow), gas bubbles (stars), extensive edema and rod-shaped bacterial clusters (arrow head) in the subcutaneous fascial tissue. **d** Necrosis in the proximal convoluted tubules (arrow), diffuse edema and rod-shaped bacterial clusters (arrow head) in the kidney. HEx200μ

Similar macroscopic findings were found in dead animals. No gross lesions were observed in fetal and uterus samples. Crepitation was noticed in the bloated areas of the goats while cutting the skin, in addition to the greenish appearance containing bubbles in the subcutaneous fascia (Fig. 1a). In the subcutaneous fascia, bubbles were seen with dark red-black colored fluid, extending from the inguinal region to the dorsal. Bleeding areas in dark red-black color in the subcutaneous region, and approximately 500–600 ml of bloody fluid in the abdominal cavity were observed. When the thoracic cavity was opened, it was found that there was a thick foamy fluid in the trachea and the lungs were edematous. Petechial hemorrhages were observed in the epicardium and endocardium (Fig. 1b).

In the microscopic examination; extensive edema, focal necrosis, gas bubbles in large vacuoles and rod-shaped bacterial clusters were detected in and around the subcutaneous fascial tissues (Fig. 1c). As a result of widespread edema, enlargement due to separation and a small number of neutrophil leukocyte infiltrations were observed in the fascial tissue and muscle fibers. Thrombi were detected in venous and capillary veins in these areas. In the lungs, pink homogeneous edema fluid was found in interlobular, interalveolar septum and alveolar lumens. In the kidney tissue, necrosis in the proximal convoluted tubulus epithelium, diffuse edema and rod-shaped bacterial clusters in the intertubular areas were observed (Fig. 1d).

**Fig. 2:**
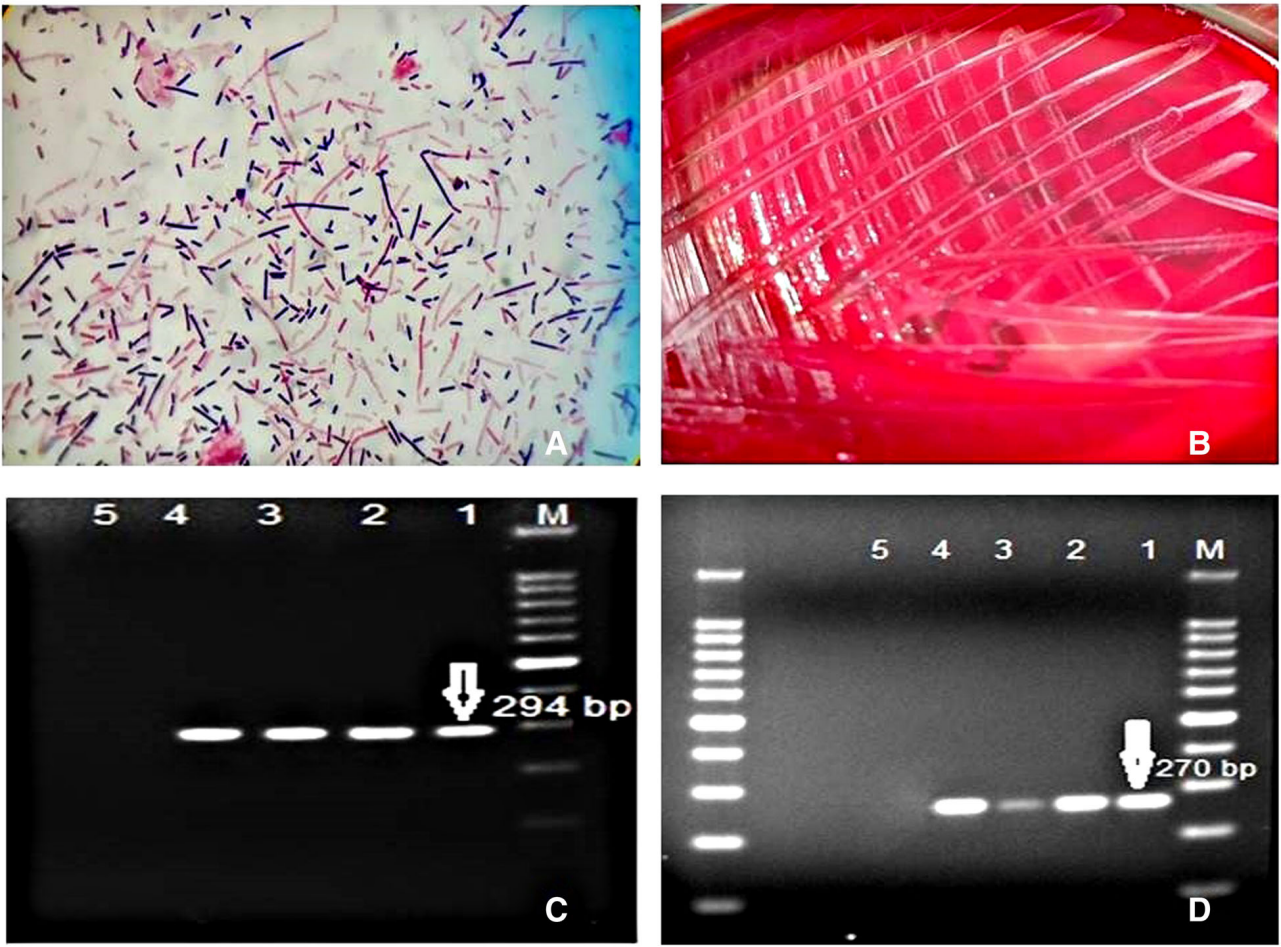
Bacteriological and PCR results obtained from edema fluid samples of three live animals. **a** Microscopic view of *C. septicum*. **b** Colony morphology of *C. septicum* in blood agar. **c** PCR amplification of *C. septicum* (294 bp). M: Marker, 1: Positive Control, 2, 3, 4: Samples, 5: Negative control. **d** PCR amplification of *C. septicum* alpha (hemolysin) toxin gene 270 bp. M: Marker, 1: Positive Control, 2, 3, 4: Samples, 5: Negative Control

Following the culture of the samples taken from nine animals under anaerobic conditions, a diffuse colony appearance as well as centrally sporulated and rod-shaped Gram positive bacteria were observed on the microscope (Fig. 2a, b). However, no growth was observed in the incubation of the samples in aerobic conditions. Flagellin gene (*fliC*) specific PCR analysis of DNA’s extracted from five pure colonies of all the samples produced positive results for *C. septicum* (Fig. 2c). Also, all the isolates were found to be positive for the alpha toxin (Hemolysin) gene in the PCR analysis (Fig. 2d). On the other hand, *C. chauvoei* could not be detected in any of the isolates by species-specific PCR.

The data obtained from one-way DNA sequence analysis of the PCR products of the flagellin gene region were aligned using the BLAST search (https://blast.ncbi.nlm.nih.gov/Blast.cgi/) with the registered *C. septicum* (HQ65058) in GenBank and the *C. botulinum* sequence (DQ844954) as an external group. Thus, a deletion at nucleotide 11 and a change in T ➔ C at nucleotide 238 were observed in our isolates when compared to the reference sequence.

## Discussion and conclusion

Gas gangrene is known as an exogenous disease in which the agent enter the body throughout skin abrasions. Because *C. septicum* is an opportunistic pathogen, it can also cause gas gangrene endogenously, especially in immunosuppressed animals [14]. No skin abrasions were noted in the clinical examination of the animals in the present study. Because most of the animals in the herd were pregnant, it was considered that the infection might be endogenous due immunosuppression caused by pregnancy. Oral administration of *C. septicum* is known to cause necrotic abomasitis in sheep, but in the necropsies of the animals examined in this study only hemorrhagic lesions were observed in abomasum [15]. *C. septicum* can be found in the intestines of healthy ruminants. It is presumed that owing to immunosuppression, it causes septicemia by entering blood circulation and then produces myonecrosis in muscles by spreading throughout the body. In the anamnesis, information was obtained that all the animals in the herd were vaccinated with a commercial polyvalent vaccine 2 months ago. The adequate immune response was probably not developed in a limited number of animals. Also, sporadic cases of gas gangrene have been reported in cattle and sheep located in the same region. It was therefore thought that the agent was likely to be present in the environment and might have been taken by these animals.

Alpha toxin production has been shown to result in subcutaneous bloating and darkening of edematous skin areas and interstitial hemorrhage in muscle tissue [16]. Detection of similar necropsy findings in animals examined here suggests that *C. septicum* isolates produced alpha toxin which was already confirmed by toxin-specific PCR. Indeed, studies have shown that *C. septicum* needs to produce alpha toxin in order to manifest specific clinical signs. Kennedy et al. (2005), reported a striking difference between alpha toxin positive and negative strains in terms of virulence [17]. The researchers observed fulminant gas gangrene symptoms within hours in mice infected with alpha toxin positive strains, whereas they did not find any clinical signs in mice that were infected with alpha toxin negative strains. Alpha toxin of *C. septicum* is structurally and functionally similar to epsilon toxin of *C. perfringens* type B and D and aerolysin of *Aeromonas hydrophila*. On the other hand, alpha toxin of *C. septicum* unlike *C. perfringens* alpha toxin, causes infiltration of immune system cells in the infection site. The histopathological finding that a large number of leukocytes were present in the lesioned area can be considered as another evidence for the production of alpha toxin or other toxins by *C. septicum* isolated in the current study. In infections caused by *C. septicum*, hemorrhage due to alpha toxin-induced microvascular destruction leads to decreased blood flow in the infection site. This ultimately leads to ischemia that will support the survival of *C. septicum* in the absence of external trauma [9, 10].

*Clostridium septicum* is generally sensitive to penicillin G, ampicillin, chloramphenicol, clindamycin, cephaloridine, oleandomycin, erythromycin, lincomycin and tetracyclines [18]. Because *C. septicum* causes sporadic disease which usually results in rapid death, there is no treatment available, in general. In this study, as the clinical signs of four affected animals had just begun which indicated the early stage of the disease, they were successfully treated with penicillin group antibiotics. This suggests that antibiotic use can be beneficial and may decrease deaths due to *C. septicum* in the early diagnosis of the disease.

*C. septicum*, commonly found in soil, has also been isolated from the feces of human and healthy animals [18]. The agent, as a postmortem invader, has the ability to rapidly spread throughout the body from the intestines of dead or agonized animals, especially ruminants. Due to the rapid spreading feature, it is possible to isolate *C. septicum* which can lead to misdiagnosis even if necropsy is made instantly following the death of animals. However, in this study, this agent was isolated from the skin lesions of live but diseased animals in addition to the samples taken at necropsy.

Although rapid diagnostic methods, such as fluorescent antibody test, are recommended for the diagnosis of gas gangrene, some drawbacks such as difficulty in obtaining commercially labeled antisera and potential complexity of the method restricts the use of this test. The fluorescent antibody test was not employed in this study due to the absence of labeled antisera. Instead, the isolates were examined for the presence of both *C septicum* and *C. chauvoei* by PCR which is a rapid and sensitive molecular method and, while the former agent was identified, the latter could not be detected at all.

In conclusion, the present study reports for the first time the isolation and identification of *C. septicum* from various samples collected in a goat herd suspected of gas gangrene. It is believed that the histopathological and molecular findings of the study overruled the possible accidental presence of the agent in these goats.

## Abbreviations

HE: Hematoxylin-eosin
PCR: Polymerase chain reaction
